# Dynamic Wrist Flexion and Extension Fatigue Induced via Submaximal Contractions Similarly Impairs Hand Tracking Accuracy in Young Adult Males and Females

**DOI:** 10.3389/fspor.2020.574650

**Published:** 2020-10-06

**Authors:** Robert I. Kumar, Garrick N. Forman, Davis A. Forman, Maddalena Mugnosso, Jacopo Zenzeri, Duane C. Button, Michael W. R. Holmes

**Affiliations:** ^1^Faculty of Applied Health Sciences, Brock University, St. Catharines, ON, Canada; ^2^Faculty of Science, University of Ontario Institute of Technology, Oshawa, ON, Canada; ^3^Robotics, Brain and Cognitive Sciences, Istituto Italiano di Tecnologia, Genoa, Italy; ^4^School of Human Kinetics and Recreation, Memorial University of Newfoundland, St. John's, NL, Canada

**Keywords:** forearm, hand, tracking, fatigue, kinematics, task performance, sex differences

## Abstract

We evaluated the effects of muscle fatigue on hand-tracking performance in young adults. Differences were quantified between wrist flexion and extension fatigability, and between males and females. Participants were evaluated on their ability to trace a pattern using a 3-degrees-of-freedom robotic manipulandum before (baseline) and after (0, 1, 2, 4, 6, 8, and 10 mins) a submaximal-intensity fatigue protocol performed to exhaustion that isolated the wrist flexors or extensors on separate days. Tracking tasks were performed at all time points, while maximal voluntary contractions (MVCs) were performed at baseline, and 2, 6-, and 10-mins post-task termination. We evaluated movement smoothness (jerk ratio, JR), shape reproduction (figural error, FE), and target tracking accuracy (tracking error, TE). MVC force was significantly lower in females (*p* < 0.05), lower than baseline for all timepoints after task termination (*p* < 0.05), with no muscle group-dependent differences. JR did not return to baseline until 10-mins post-task termination (most affected), while FE returned at 4-mins post-task termination, and TE at 1-min post-task termination. Males tracked the target with significantly lower JR (*p* < 0.05), less TE (*p* < 0.05), and less FE (*p* < 0.05) than females. No muscle group-dependent changes in hand-tracking performance were observed. Based on this work, hand tracking accuracy is similarly impaired following repetitive submaximal dynamic wrist flexion or extension. The differences between male and female fatigability was independent of the changes in our tracking metrics.

## Introduction

During a submaximal isometric dual task paradigm of the distal upper-limb (concurrent hand-gripping and wrist flexion/extension), the wrist flexors exhibit task-specific characteristics (i.e., wrist flexor muscle activity changes to a large extent with changes in task parameters) (Mogk and Keir, 2003; Forman et al., 2019; Forman G. N. et al., 2020). In contrast, the wrist extensors act as joint stabilizers (Holmes et al., 2015) and exhibit consistently high levels of muscle activity regardless of changes to dual task parameters (Mogk and Keir, 2003; Forman et al., 2019; Forman G. N. et al., 2020). As a whole, the wrist extensor muscles have a lower force generating capacity than the flexors (Singh and Karpovich, 1968), and this is independent of wrist posture (La Delfa et al., 2015). Only at full wrist extension (max range) have the extensors been shown to generate a greater wrist moment than the flexors (Hallbeck, 1994). This reduced wrist extensor strength is thought to predispose the wrist extensor muscles to an earlier onset of impaired movement resulting from fatigue (Hägg and Milerad, 1997) since the extensor muscles operate at a greater percentage of maximum to balance joint moments, which could lead to an increased risk of overuse injury (Shiri and Viikari-Juntura, 2011). Given the task specificity in muscular roles, there has been a lack of investigation into how flexor or extensor fatigue may independently influence hand control.

While the primary functions of the forearm muscles are well-established, the effects of forearm fatigue on hand task performance are less understood. Performance fatigability is a decline in the objective measures of performance as a result of a fatiguing protocol (Enoka and Duchateau, 2016). In joints where fatigue and performance have been examined, performance fatigability may manifest as decreased movement accuracy (Mugnosso et al., 2019) and increased force variability (Missenard et al., 2008a). Some mechanisms responsible for the decline in performance are due to peripheral (i.e., contractile function) or central (i.e., muscle activation) factors. Recently, we examined the effects of sustained isometric contractions on hand-tracking accuracy (Forman D. A. et al., 2020). Participants performed either sustained maximal isometric wrist flexion or extension until they were unable to maintain 25% of their original maximal force. Participants performed a hand-tracking task pre- and post-task termination using a 3-degrees-of-freedom manipulandum. We found that hand tracking accuracy was significantly worse than baseline immediately post-task termination but recovered within 1-min. Surprisingly, there were no differences in tracking error metrics between the flexion and extension sessions. Because fatigue was induced through maximal isometric contractions, generalizing these findings to performance fatigue induced by dynamic contractions is difficult.

There are clear differences between isometric and dynamic contractions (i.e., changes in muscle length, moment arms and motor unit discharge rate), therefore, performance fatigability could be impaired differently between contraction modalities (Hunter, 2018). Dynamic contractions have been reported to prompt greater deficits in force steadiness (Lavender and Nosaka, 2006) and fatigue resistance (Yoon et al., 2013) compared to isometric contractions. Given our understanding of the force-velocity relationship, it may be speculated that dynamic contractions induce fatigue earlier due to the increased time spent under eccentric tension at which the external load is optimally leveraged against muscular effort (i.e., resisting higher intensities due to mechanical disadvantage). At the neuromuscular level, it has been shown that motor unit discharge rates are greater during dynamic contractions compared to isometric (Kallio et al., 2013). Finally, there is also evidence that eccentric contractions induce the greatest joint position sense impairments (Brockett et al., 1997). Additionally, the intensity of the contraction will impact the severity of fatigue (Enoka and Duchateau, 2016; Hunter, 2018). In a study with submaximal dynamic fatiguing contractions (70% MVC) of the shoulder flexors, performance was maintained but with an altered movement strategy (Côté et al., 2005). Further, another group investigated the effects of sustained submaximal wrist extension contractions (15% MVC) on mouse-tracking performance and found a decrease in tracking accuracy (Huysmans et al., 2008). The mechanisms that lead to decreased task performance after submaximal and maximal contractions are both central and peripheral in nature (Gandevia, 2001; Allen et al., 2008). Namely, peripheral fatigue is with reference to deleterious, exercise-induced processes that impair force output or performance from the neuromuscular junction and further distally. Central fatigue is specific to alterations that occur proximal to the neuromuscular junction (i.e., spinal and supraspinal centres) and are often associated with decrements in voluntary activation (Taylor and Gandevia, 2008; Taylor et al., 2016). There is an enhanced effect of central fatigue following submaximal contractions, and these changes are often a result of decreased excitatory input, increased inhibitory input and a decrease in responsiveness of motoneurons (Taylor and Gandevia, 2008; Taylor et al., 2016). On the other hand, peripheral fatigue has been associated with increases in exercise intensity (i.e., resulting from maximal contractions) (Thomas et al., 2016). One particular peripheral mechanism is low-frequency fatigue, which occurs when greater amounts of Ca^2+^ are required to perform the same task, ultimately modifying the Ca^2+^-force relationship (Edwards et al., 1977; Bruton et al., 1998). Additionally, decreased resting muscle twitch force has been observed as a result of peripheral fatigue (Søgaard et al., 2006). Søgaard et al. (2006) found that voluntary drive decreased (central factor) concurrently with a decrease in resting muscle twitch force throughout the 43-min submaximal contraction of the elbow flexors. However, the direct effect of a submaximal dynamic fatigue task on tracking accuracy is largely unexplored.

Greater fatigue resistance in females has been reported for various muscle groups during isometric and dynamic fatigue protocols of various intensities (Hunter and Enoka, 2001; Avin et al., 2010; Svendsen and Madeleine, 2010; Yoon et al., 2015; Hunter, 2016). This disparity in fatigue resistance has even been found in females compared to males when matched for strength (Fulco et al., 1999). Some of the proposed physiological mechanisms for these observed sex-differences include higher relative proportions of type I muscle fiber distribution within each muscle, decreased glycolytic activity and decreased activation of group III and IV afferent inhibition in females (Hunter, 2018). To our knowledge, sex differences in forearm fatigue has been minimally studied, although some work has been completed on force steadiness (Brown et al., 2010) and forearm and wrist strength across various postures (La Delfa et al., 2015). Notably, Brown et al. (2010) found that males were steadier than females in a wrist flexion task across various wrist postures. Further, La Delfa et al. (2015) demonstrated that forearm rotation affects males and females differently. Given known sex differences for fatigue resistance and force steadiness, generalized findings from male-specific research at the wrist may not be applicable to females.

The purpose of this study was to examine how a submaximal dynamic fatigue protocol with isolated wrist flexor or extensor fatigue would influence hand-tracking accuracy using a wrist robot. A secondary purpose was to examine fatigue-related tracking accuracy differences between sexes, given the greater fatigue resistance reported in females for various muscle groups during isometric and dynamic fatigue protocols. We hypothesized that a larger deficit would be observed in all tracking metrics for the extension fatigue session compared to the flexion fatigue session due to known anatomical and functional differences between the flexors and extensors. This was not observed in our previous study on an isometric fatiguing task, which may have been due to the previous isometric fatiguing protocol being matched with a dynamic task, whereas the present study matches a dynamic fatigue protocol with a dynamic task (Forman D. A. et al., 2020). We also hypothesized that males would be more proficient at tracking than females due to the reported sex differences for force steadiness (Brown et al., 2010; Jakobi et al., 2018).

## Methods

### Participants

Eighteen young, recreationally active adults were recruited: 8 females (Height: 168 ± 6 cm, Weight: 67 ± 14 kg, Age: 23 ± 2 years) and 10 males (Height: 179 ± 9 cm, Weight: 81 ± 12 kg, Age: 25 ± 3 years). Our sample size was similar to previous groups investigating comparable outcome measures, including Forman D. A. et al. (2020), Huysmans et al. (2008), Emge et al. (2013), Missenard et al. (2008a,b), and Jaric et al. (1997, 1999). All participants were self-reported right-hand dominant and free from known neurological or musculoskeletal disorders of the upper extremities. This study received ethics clearance by the Research Ethics Board at Brock University (REB: #16-263). Participants read and signed the informed consent prior to beginning any experimental procedures. Participants visited the lab on 3 separate occasions, where the two experimental sessions were separated by a minimum of 6 days (order of the two sessions were randomized).

### Experimental Procedure

#### Day 1: Determination of Load for Fatiguing Protocol

**Figure 1:**
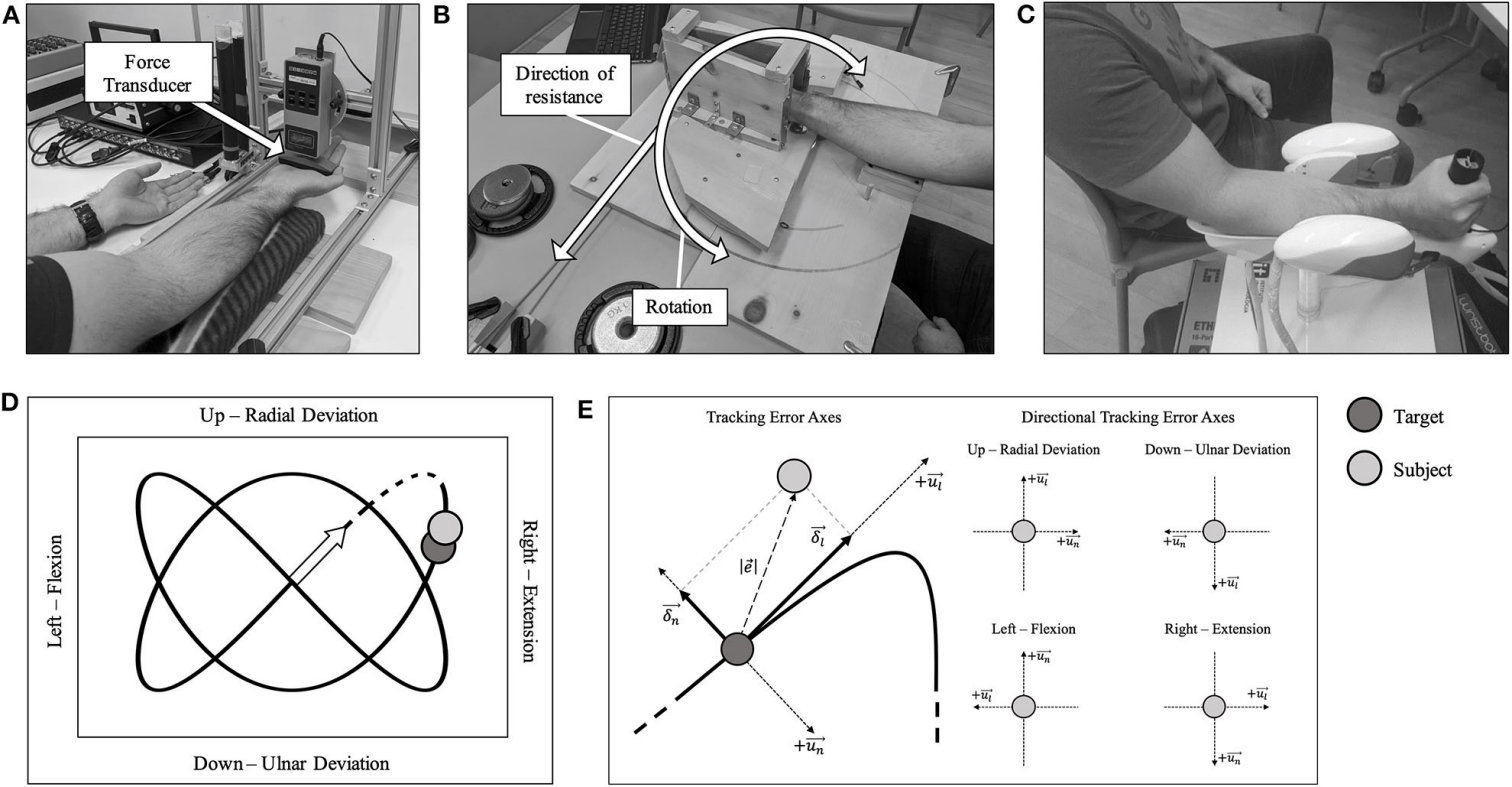
**(A)** Participants sat comfortably in front of the force transducer with their left-hand palm-up on the table. **(B)** Custom-made dynamic fatigue apparatus. **(C)** A participant seated with their forearm and hand placed in the WristBot. **(D)** A 2:3 Lissajous curve, which was visible during the hand-tracking. Movement directions are shown with their associated kinesiological action. The origin is shown as an arrow. The dashed portion of the curve was excluded from all analyses. **(E)** Left: the overall tracking error axes are shown. Right: the specific directional tracking error axes are shown. All components are with respect to the kinesiological action. The longitudinal axis is represented by $+\vec{u_{l}}$, with its respective component represented by δ_*l*_. The normal axis is represented by $+\vec{u_{n}}$, with its respective component represented by δ_*n*_. The Euclidean distance between the subject's handle (*H*—light gray) and the target (*T*—dark gray) as $\left|\vec{e}\right|$.

Participants performed a graded submaximal dynamic wrist flexion and extension protocol using their dominant hand, to determine the fatiguing weight for the following experimental sessions. The custom apparatus used to determine the fatiguing load was also used for the fatiguing protocol (Figure 1B). Participants were familiarized with the protocol by warming up with a low resistance (1 kg) with a controlled cadence. All movement began in maximum flexion for flexion fatigue sessions and maximum extension for extension fatigue sessions and took 2 s to reach the end-range position for the respective movement (2 s for concentric movement, 2 s for eccentric movement, for a total of 4 s per repetition). Each set was separated by 5 min of rest to mitigate fatiguing effects. The weight was progressively increased at each set until participants could perform 15 repetitions (but not exceed 20) to the cadence defined above. Set termination was determined by the investigators when the participant could no longer reach the half-way point of the flexion/extension movement. The protocol was repeated for the other muscle group after 5 min of rest. The purpose of determining the fatigue loads in advance was to have a target weight for days 2 and 3 to ensure all participants were able to achieve 15–20 repetitions in an effort to systematically control for the rate of fatigue between the two sessions. At the end of day 1, we knew the participant specific weight required for the day 2 and 3 flexion/extension fatigue protocol sessions.

#### Days 2 and 3: Flexion or Extension Fatigue

##### Maximal voluntary contractions

Participants sat in front of a table-mounted force transducer (Model: BG 500, Mark-10 Corporation, New York, USA), with their right forearm resting on a padded surface that allowed their hand and wrist to hang freely. The hand was positioned so that the force was applied along the metacarpophalangeal joints (Figure 1A), and this position was marked with indelible ink to ensure consistency for the duration of the trial. The participant's left hand was placed on the table (shown in Figure 1A) with the palm facing up to prevent any assistance during the MVC from the contralateral limb. For wrist flexion MVCs, the forearm was supinated, and for wrist extension MVCs, the forearm was pronated. Participants performed 2 maximal voluntary contractions (MVCs), each held for ~3–4 s, with 1-min inter-trial rest. Participants were able to concurrently see their force value increase during the duration of each MVC trial and knew what their peak MVC value was after each trial. Verbal encouragement was provided for all MVCs. The MVC with the higher force was determined as the true MVC force. In the case that a MVC was not deemed maximal, either by the participant or by the investigator, a third MVC was performed after adequate rest.

##### Tracking task and fatigue protocol

The tracking task was performed using an end-effector robotic device known as the WristBot (Figure 1C, Istituto italiano di Tecnologia, Genoa, Italy) (Iandolo et al., 2019). The device is a 3-degrees-of-freedom manipulandum with the following ranges of motion: Flexion/Extension = ±62°; Radial/Ulnar Deviation = +45°/−40°; Pronation/Supination = ±60°. The goal of the task was to track a yellow target as it traversed a set pattern (2:3 Lissajous curve), on a computer screen at eye-level (Figure 1D). The participant's position was represented by a blue target, and they were instructed to overlay their position as best as possible with the target. Participants moved the WristBot handle using a combination of up and down (radial and ulnar deviation, y-axis) and side to side (flexion and extension, x-axis) movements. Pronation/supination movement was restricted. One tracking trial took 20 s to complete. They were familiarized with the WristBot tracking task by performing 12 practice trials, which we found to be adequate to learn the task (Forman D. A. et al., 2020). For both the fatigue protocol and tracking task, elbow and shoulder joint angles were matched between sessions. There was 5-min of rest between practice trials and the 5 baseline tracking trials.

**Figure 2:**
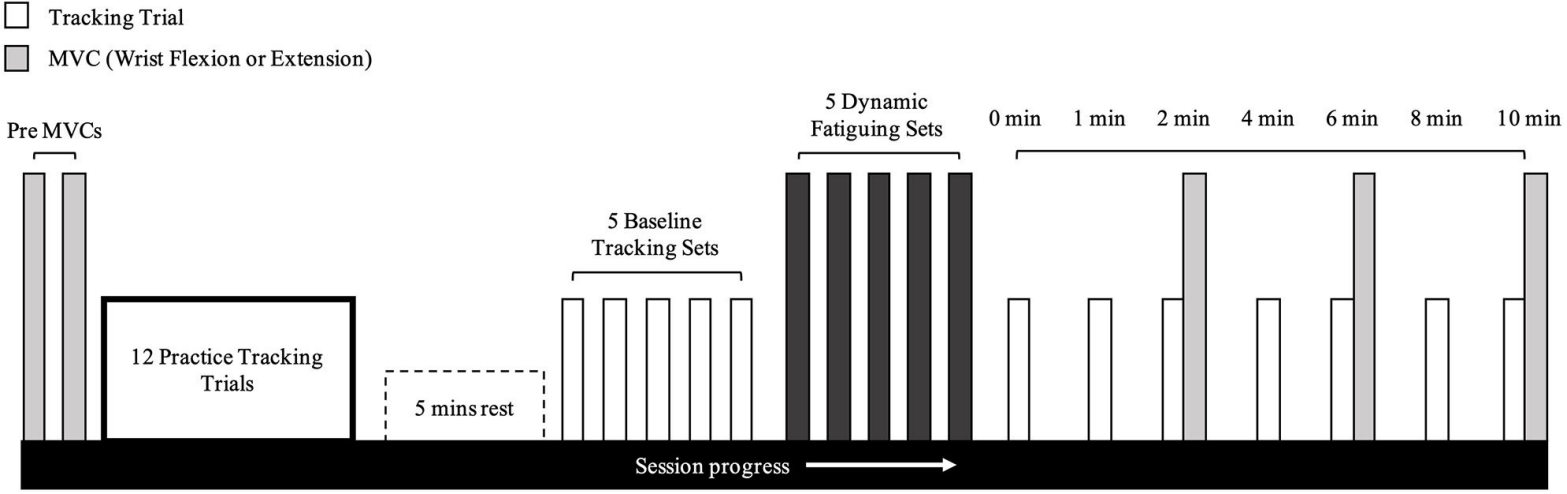
Timeline of experimental protocol. This protocol was the exact same for wrist flexion and extension fatigue days.

Once baseline tracking trials were completed, the participant was seated comfortably at the dynamic fatigue device (Figure 1B). The participant placed their hand in the center of the dynamic fatigue apparatus with a neutral wrist posture, and we matched the axis of rotation of their wrist approximately to the axis of rotation of the apparatus. The resistive load was attached with a rope and wrapped around a pulley to counter concentric movement. Participants performed 5 sets to a strict cadence (4 s per repetition, as detailed above), using the load determined on day 1. They were instructed to perform repetitions to failure and to move to their maximum active wrist range of motion. Participants must have passed half-way (returning to a neutral wrist posture-−0° of wrist flexion-extension) on the custom-built apparatus for a repetition to be valid. Once the participant could no longer pass the half-way point, the set was terminated (see Table 1 for the resistances and repetitions). Forty five seconds rest was provided between each set. Upon completion of the final set, the participant immediately transitioned to the WristBot to perform the post-task termination tracking trials (Figure 2). The transition was made as quickly as possible (< 10 s) and the recovery time began measurement immediately after they were positioned in the WristBot. The participants performed tracking trials at 0-, 1-, 2-, 4-, 6-, 8-, and 10-min post-task termination. MVCs were performed directly after the tracking task at min 2, 6, and 10 to evaluate recovery in maximal force production.

**Table 1:** Resistance used for males and females during the flexion and extension fatigue task and the number of repetitions achieved for each of the 5 sets.

	**Day**	**Weight**	**Set 1**	**Set 2**	**Set 3**	**Set 4**	**Set 5**
		**(kg)**	**(repetition #)**	**(repetition #)**	**(repetition #)**	**(repetition #)**	**(repetition #)**
**Females**^†^	Flexion	2.40 (0.30)#	19.4 (5.9)	9.0 (1.7)^*^	8.0 (2.1)^*^	5.9 (1.5)^*^	6.6 (1.9)^*^
	Extension	1.57 (0.26)	16.7 (3.8)	8.3 (1.4)^*^	7.1 (2.0)^*^	6.7 (2.0)^*^	7.0 (2.7)^*^
**Males**^†^	Flexion	4.26 (0.83)#	18.9 (4.6)	8.7 (2.3)^*^	6.6 (1.0)^*^	5.4 (1.3)^*^	4.5 (0.7)^*^
	Extension	2.35 (0.36)	17.4 (3.2)	7.7 (4.2)^*^	5.9 (2.5)^*^	5.3 (0.8)^*^	5.4 (1.3)^*^

*All values are presented as a mean (standard deviation)*.

* * = pooled data are significantly different from Set 1*;

† * = data are significantly different between sexes; # = data are significantly different between muscle groups*.

### Data Analysis

All kinematic data from the WristBot were collected at 100 Hz and analyzed offline using MATLAB (MathWorks Inc., Natick, MA, USA). A 6th-order Savitzky-Golay filter was applied to smooth the kinematic data using a 170 ms window to mimic an 11 Hz low-pass filter (Squeri et al., 2010). The initial movement during each trial (dashed portion in Figure 1D) was excluded from analysis since there was no starting cue and participants were often caught off-guard by the initial movement of the target. We incorporated several measures to quantify tracking performance and movement smoothness during each trial. For simplicity, see Figure 1E for a visual representation of the directions of movement and their respective axes.

#### Tracking Error

This metric was used to quantify the mean Euclidean distance (Equation 1) between the subject's position and the target's position (Squeri et al., 2010):

(1) $$\begin{array}{l} |\vec{e}|=\frac{\sum_{i=1}^{N}\sqrt{{\left(H_{x}-T_{x}\right)}^{2}+{\left(H_{y}-T_{y}\right)}^{2}}}{N} \end{array}$$

where *H* and *T* represent the handle and target in the x- and y-directions. Tracking error was assessed as a mean over *N* datapoints within each tracking trial. Directional tracking error was assessed for flexion, extension, radial-, and ulnar-deviation.

#### Longitudinal Component of Tracking Error

The longitudinal and normal components of tracking error are orthogonal (Figure 1E, left). This component was used to assess how far ahead or behind the subject was relative to the target. A positive longitudinal component represents the subject being ahead of the target, and a negative value represents the subject behind the target. Equations 2 and 3 were used to determine the longitudinal component (Squeri et al., 2010):

(2) $$\begin{array}{l} \vec{u_{l}}=\frac{1}{\sqrt{{Ṫ}_{x}^{2}+{Ṫ}_{y}^{2}}}\left[\begin{array}{c} {Ṫ}_{x}\\ {Ṫ}_{y} \end{array}\right]=\left[\begin{array}{c} ux_{l}\\ uy_{l} \end{array}\right] \end{array}$$

(3) $$\begin{array}{l} \delta_{l}=\vec{e}\cdot \vec{u_{l}} \end{array}$$

where Equation 2 represents the unit vector of the trajectory of the target (*T*) for each data point, and Equation 3 represents the longitudinal component of the tracking error. The first derivative of the target displacement was used to find the tangent vector to the trajectory and is normalized to calculate the unit vector. The direction is given in Equation 3 by multiplying the respective trial's tracking error $\vec{e}$ with the unit vector, $\vec{u_{l}}$, from Equation 2. The solution of Equation 3 provides a positive (handle ahead of target) or a negative (handle behind target) product.

#### Normal Component of Tracking Error

The longitudinal component explained whether the subject was tracking ahead or behind the target and is paired with the normal component, which describes if the participant is to the right or left of the target tangent trajectory (Squeri et al., 2010):

(4) $$\begin{array}{l} \vec{u_{n}}=\left[\begin{array}{c} {uy}_{l}\\ {-ux}_{l} \end{array}\right] \end{array}$$

(5) $$\begin{array}{l} \delta_{n}=\vec{e}\cdot \vec{u_{n}} \end{array}$$

where Equation 4 represents the unit vector of the normal component, and Equation 5 gives the normal component.

#### Figural Error

This metric was used to quantify how well the participant reproduced the shape of the trajectory (Equation 6), and is insensitive to the speed of the movement (Squeri et al., 2010):

(6) $$\begin{array}{l} dist_{A-B}\left(i\right)=\|A_{i}-B_{j}\|i=1,\;2,\ldots n\\ dist_{B-A}\left(j\right)=\|A_{i}-B_{j}\|j=1,\;2,\ldots m\\ FE_{AB}=\frac{\sum_{i=1}^{n}dist_{A-B}\left(i\right)+\sum_{j=1}^{m}dist_{B-A}\left(j\right)}{n+m} \end{array}$$

where the vector *dist*_*A*−*B*_ represents the Euclidean distance between the trajectory B and each point of A. The inverse is true for vector *dist*_*B*−*A*_. Values *n* and *m* represent each point in the two vectors. If the figural error score was 0, the participant perfectly overlaid their path over the target path during a trial.

#### Jerk Ratio

Jerk is the 3rd derivative of position, and was calculated as the integrated squared jerk (ISJ, Equation 7) (Platz et al., 1994). Equation 8 is the jerk ratio:

(7) $$\begin{array}{l} ISJ=\int_{0}^{d}\left({\overset{...}{H}}_{x}^{2}+{\overset{...}{H}}_{y}^{2}\right)dt \end{array}$$

(8) $$\begin{array}{l} JR=\frac{ISJ_{H}}{ISJ_{T}} \end{array}$$

where *d* is the duration of the tracking trial, and *H* is the movement of the subject in the x- and y-directions. *ISJ*_*H*_ represents subject jerk, while *ISJ*_*T*_ represents target jerk. A value of 1 represents a movement that is perfectly smooth with respect to the target, while anything > 1 is less smooth.

### Statistics

All data were examined across the tracking trace after the dashed portion in Figure 1D and were also divided into the four movement directions. All statistical analyses were performed using SPSS (SPSS, IBM Corporation, Armonk, NY, USA). A two-way ANOVA (Load [Flexion/Extension] × Sex [Male/Female]) was used to evaluate differences for absolute loads between males and females. A three-way repeated measures ANOVA (Session [Flexion/Extension day] × Sex [Male/Female] × Set [1, 2, 3, 4, 5]) was computed for the number of repetitions during the fatiguing protocol. A three-way repeated measures ANOVA (Session [Flexion/Extension day] × Sex [Male/Female] × Measurement Time [Baseline, 0, 1, 2, 4, 6, 8-, and 10-min post-task termination]) was used for MVC force, tracking error, the longitudinal and normal components, figural error and jerk ratio. The Greenhouse-Geisser correction was used for interpretation where Mauchly's test of sphericity was violated. Main effects and interactions were followed up with *post-hoc* analyses and pairwise comparisons using Bonferroni corrections. All significance levels were set at *p* < 0.05. Effect sizes were calculated as partial eta squared ($\eta_{\text{p}}^{2}$) for ANOVAs, Cohen's *d* for pairwise comparisons where mean variances were equal, Glass' delta (Δ) where mean variances were unequal, and Hedges' *g* where sample sizes were unequal (exclusively for the load used between sexes [8F, 10M]) (Ialongo, 2016).

## Results

### Load and Repetitions During the Fatigue Protocol

A two-way interaction was observed between sex and muscle group [*F*_(1,16)_ = 84.323, *p* = 0.002, $\eta_{\text{p}}^{2}$ = 0.455]. *Post-hoc* analyses revealed that males had higher loads for flexion [*F*_(1,16)_ = 36.228, *p* < 0.001, *g* = 2.855] and extension [*F*_(1,16)_ = 25.528, *p* < 0.001, *g* = 2.396] when compared to females. Additionally, flexion loads were higher than extension loads for males [*F*_(1,9)_ = 68.144, *p* < 0.001, *d* = 2.987] and for females [*F*_(1,7)_ = 25.622, *p* = 0.001, *d* = 2.717].

There were no significant differences for number of repetitions during the fatigue protocol between males and females [*F*_(1,16)_ = 0.904, *p* = 0.356, $\eta_{\text{p}}^{2}$ = 0.053]. Additionally, no main effect was observed between muscle groups [*F*_(1,16)_ = 2.704, *p* = 0.120, $\eta_{\text{p}}^{2}$ = 0.145]. A main effect was observed for the fatiguing protocol sets [*F*_(1.561,24.970)_ = 139.658, *p* < 0.001, $\eta_{\text{p}}^{2}$ = 0.897; Table 1]. Pairwise comparisons for the fatiguing protocol sets reveal that set 1 had a significantly higher number of repetitions than set 2 (*p* < 0.001, Δ = 2.198), set 3 (*p* < 0.001, Δ = 2.568), set 4 (*p* < 0.001, Δ = 2.836) and set 5 (*p* < 0.001, Δ = 2.829).

### MVC Force

**Figure 3:**
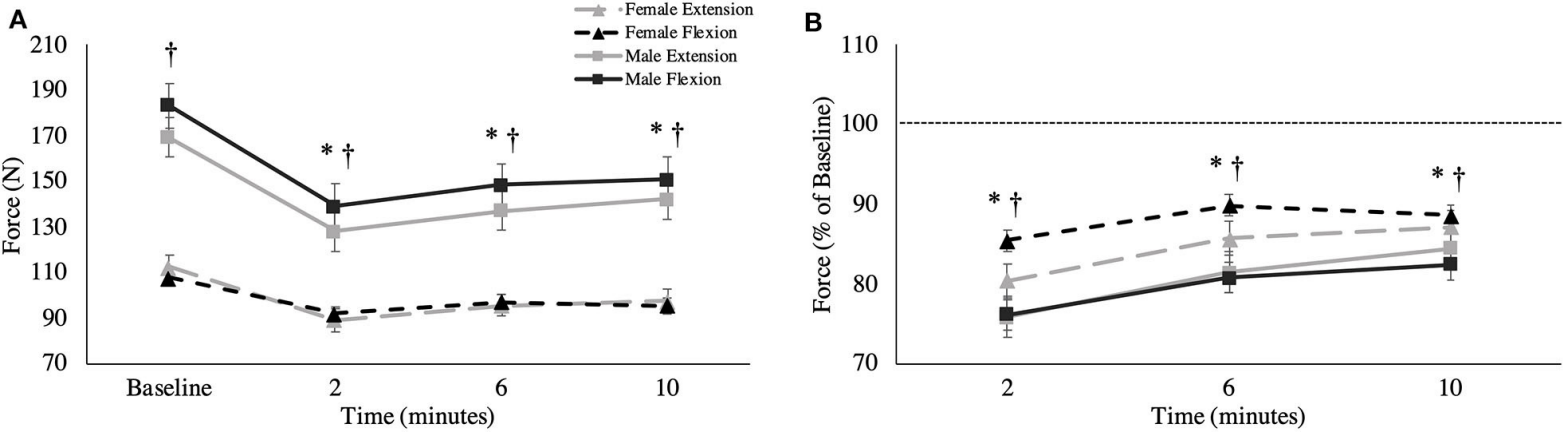
**(A)** Absolute MVC force (newtons) and **(B)** relative MVC force across all time points. This is shown as group averages (with standard error bars). The data is separated by sex and muscle group. ^†^ = data are significantly different between sexes; * = pooled data are significantly different from baseline.

An interaction was observed between sex and time [*F*_(1.399,22.380)_ = 9.372, *p* = 0.003, $\eta_{\text{p}}^{2}$ = 0.369] where males fatigued more than females. At 2-min post-task termination, female force was significantly reduced by 14.5% of MVC for flexion (*p* = 0.005, *d* = 1.153) and 19.6% of MVC for extension (*p* = 0.022, *d* = 1.189), while male force was reduced by 23.8% of MVC for flexion (*p* < 0.001, Δ = 1.349) and 24.1% of MVC for extension (*p* = 0.002, Δ = 0.967). MVC force did not return to baseline by 10-min post-task termination, regardless of sex (Figure 3). At 10-min post-task termination, males recovered from their respective force deficit by 6.3% of MVC for flexion and 8.6% of MVC for extension, while females recovered by 3.1% of MVC in flexion and 6.8% of MVC in extension. Surprisingly, no significant absolute differences were observed between MVC forces between the flexors and extensors [*F*_(1,16)_ = 1.043, *p* = 0.322, $\eta_{\text{p}}^{2}$ = 0.061].

### Tracking Error

#### Overall Tracking Error

**Figure 4:**
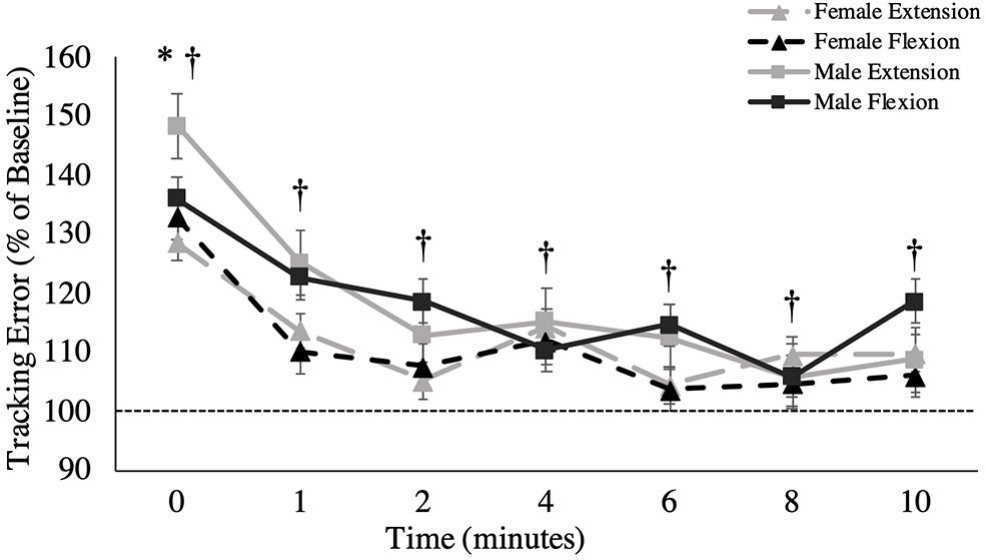
Overall tracking error (percentage of baseline) across recovery time points. The dashed line represents baseline tracking error. This metric is presented as group averages (with standard error bars). ^†^ = data are significantly different between sexes; * = pooled data are significantly different from baseline.

**Figure 5:**
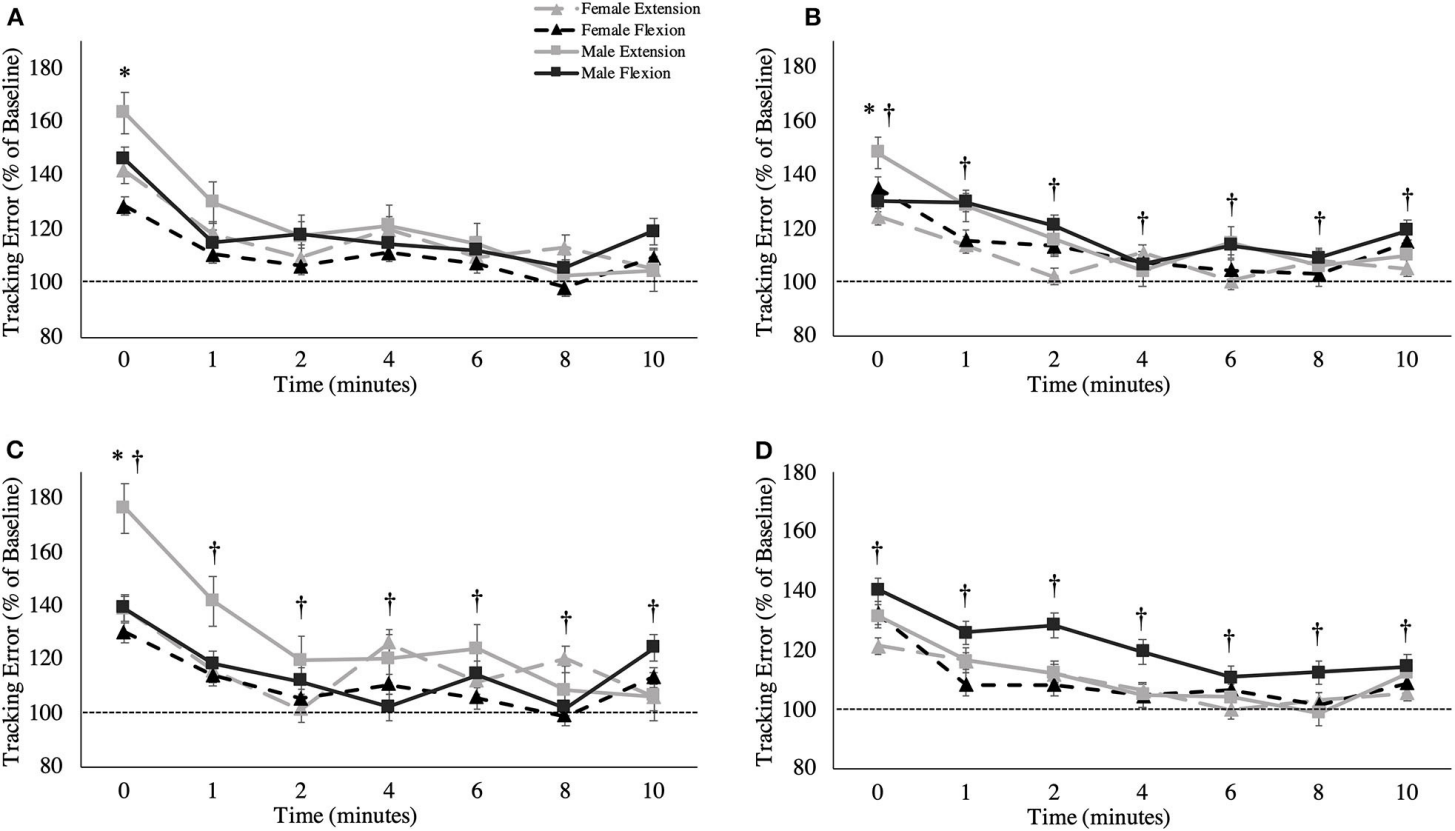
Tracking error (percentage of baseline) across recovery time points for specific movement directions: **(A)** ulnar deviation, **(B)** radial deviation, **(C)** extension movement, and **(D)** flexion. The dashed line represents baseline tracking error. This metric is presented as group averages (with standard error bars). The data is separated by sex and muscle group. ^†^ = data are significantly different between sexes; * = pooled data are significantly different from baseline.

A main effect of sex was observed for overall tracking error [*F*_(1,16)_ = 6.109, *p* = 0.025, $\eta_{\text{p}}^{2}$ = 0.276; Figure 4]. Males stayed closer to the target compared to females during the tracking task, independent of fatigue (Males: 1.40 ± 0.16°; Females: 1.74 ± 0.15°). When tracking error was broken down into the four distinct directions of movement, main effects of sex were observed for radial deviation [*F*_(1,16)_ = 8.053, *p* = 0.012, $\eta_{\text{p}}^{2}$ = 0.335; Figure 5B], extension [*F*_(1,16)_ = 4.798, *p* = 0.044, $\eta_{\text{p}}^{2}$ = 0.231; Figure 5C] and flexion [*F*_(1,16)_ = 5.238, *p* = 0.038, $\eta_{\text{p}}^{2}$ = 0.247; Figure 5D], but not for ulnar deviation [*F*_(1,16)_ = 3.123, *p* = 0.096, $\eta_{\text{p}}^{2}$ = 0.163]. Main effects for time were also observed for overall tracking error [*F*_(2.703,43.254)_ = 10.331, *p* < 0.001, $\eta_{\text{p}}^{2}$ = 0.392; Figure 4], ulnar deviation [*F*_(2.408,38.524)_ = 9.118, *p* < 0.001, $\eta_{\text{p}}^{2}$ = 0.363; Figure 5A], radial deviation [*F*_(7,112)_ = 5.758, *p* = 0.001, $\eta_{\text{p}}^{2}$ = 0.265; Figure 5B], extension [*F*_(2.478,39.642)_ = 8.159, *p* = 0.001, $\eta_{\text{p}}^{2}$ = 0.338; Figure 5C], and flexion [*F*_(3.449,55.187)_ = 5.991, *p* = 0.05, $\eta_{\text{p}}^{2}$ = 0.272; Figure 5D] directions of movement of the wrist. *Post-hoc* testing for overall tracking error and specific movement directions *except* flexion revealed that tracking error was significantly greater from baseline only when measured immediately post-task termination (time point 0).

#### Longitudinal Component of Tracking Error

**Figure 6:**
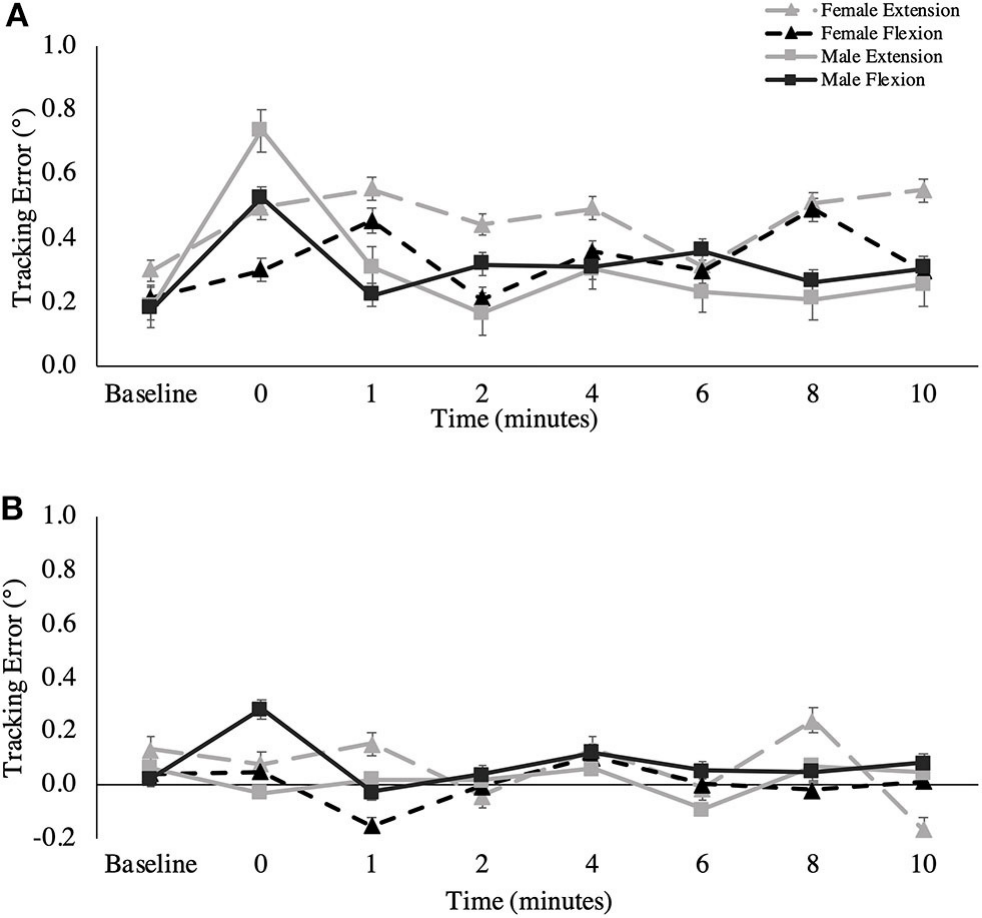
**(A)** Longitudinal and **(B)** normal component of tracking error (degrees) across all recovery time points. This metric is presented as group averages (with standard error bars). The data is separated by sex and muscle group.

Figure 6A shows the overall longitudinal component of tracking error across all time points. A main effect for fatigue session was observed for the longitudinal component of tracking error in the extension direction of wrist movement. Subjects were farther ahead of the target in the extension direction after extensor fatigue, compared to after flexor fatigue [Extension fatigue: 0.38 ± 0.62°; Flexion fatigue: 0.21 ± 0.68°, *F*_(1,16)_ = 5.421, *p* = 0.033, $\eta_{\text{p}}^{2}$ = 0.253]. Main effects of time were observed for the longitudinal component of tracking error in the extension [*F*_(3.541,56.653)_ = 3.278, *p* = 0.021, $\eta_{\text{p}}^{2}$ = 0.170] and radial direction [*F*_(3.120,49.915)_ = 2.798, *p* = 0.048, $\eta_{\text{p}}^{2}$ = 0.149] of wrist movement, however, pairwise comparisons did not reveal specific differences between any given post-task termination time point for either movement direction.

#### Normal Component of Tracking Error

Figure 6B shows the overall normal component of tracking error across all time points. A two-way interaction was observed for time and session for the overall normal component of tracking error [*F*_(4.371,69.937)_ = 2.818, *p* = 0.028, $\eta_{\text{p}}^{2}$ = 0.150], as well as for the ulnar direction of movement [*F*_(3.511,56.182)_ = 3.836, *p* = 0.011, $\eta_{\text{p}}^{2}$ = 0.193]. For the overall movement, *post-hoc* testing revealed a significant difference only on the flexion fatigue day between 1 and 4-min post-task termination (1-min: −0.05 ± 0.36°; 4-min: 0.17 ± 0.34°, *p* = 0.019, Δ = 0.976), where participants were farther to the right of the target at 4 min. For ulnar specific movement, there were significant differences between 0 and 4-min post-task termination on the extension fatigue day, (0-min: −0.26 ± 0.38°, 4-min: 0.07 ± 0.24°, *p* = 0.049, Δ = 0.873), and between 1 and 4-min post-task termination on the flexion fatigue day (1-min: −0.008 ± 0.34°, 4-min: 0.181 ± 0.31°, *p* = 0.037, Δ = 0.560). For the radial direction of wrist movement, a main effect was observed for session, where participants exhibited a greater bias to the right on the wrist extension day, which is the positive normal component of the radial direction [Extension fatigue: 0.09 ± 0.34°; Flexion fatigue: −0.01 ± 0.29°, *F*_(1,16)_ = 6.355, *p* = 0.023, $\eta_{\text{p}}^{2}$ = 0.284]. A main effect was also observed for time for the radial normal component [*F*_(3.858,61.725)_ = 4.732, *p* = 0.002, $\eta_{\text{p}}^{2}$ = 0.228]. Pairwise Bonferroni comparisons revealed specific differences between 0-min post-task termination and 2-min post-task termination (0-min: 0.22 ± 0.38°, 2-min: −0.07 ± 0.31°, *p* = 0.002, Δ = 0.784), as well as between 2-min post-task termination and 4-min post-task termination (2-min: −0.07 ± 0.31°, 4-min: 0.09 ± 0.28°, *p* = 0.037, Δ = 0.548). A two-way interaction was observed between muscle and sex for the normal component of tracking error for the extension direction [*F*_(1,16)_ = 6.604, *p* = 0.021, $\eta_{\text{p}}^{2}$ = 0.292], but main effects for muscle and for sex were non-significant.

#### Jerk Ratio

**Figure 7:**
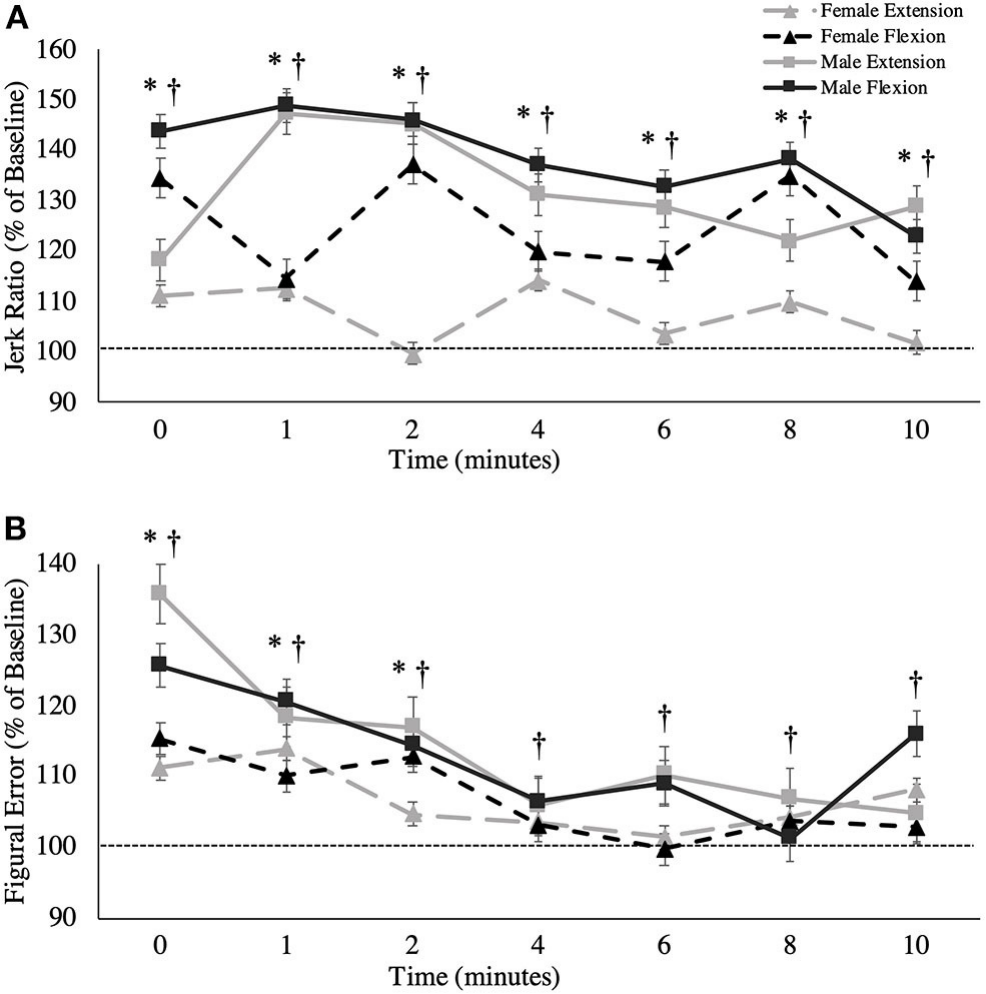
**(A)** Jerk ratio (percentage of baseline) and **(B)** figural error (percentage of baseline) across all time points. This metric is presented as group averages (with standard error bars). The dashed line represents baseline error. The data is separated by sex and muscle group. ^†^ = data are significantly different between sexes; * = pooled data are significantly different from baseline.

A main effect was observed between sexes, where males exhibited less jerk than females irrespective of the fatigue condition across all trials, including baseline [Males: 112.52 ± 13.36; Females: 152.99 ± 13.13, *F*_(1,16)_ = 12.358, *p* = 0.003, $\eta_{\text{p}}^{2}$ = 0.436; Figure 7A]. Additionally, a main effect of time was observed for the jerk ratio [*F*_(3.774,60.388)_ = 5.919, *p* = 0.001, $\eta_{\text{p}}^{2}$ = 0.270]. Pairwise comparisons revealed that this elevation in jerk ratio was substantial between the following timepoints in contrast to baseline: 0-min (*p* = 0.011, Δ = 0.790), 1-min (*p* = 0.003, Δ = 0.869), 2-min (*p* = 0.001, Δ = 0.903), 4-min (*p* = 0.009, Δ = 0.742), 6-min (*p* = 0.0017, Δ = 0.585), and 8-min (*p* = 0.023, Δ = 0.771).

#### Figural Error

A main effect of sex was found for figural error, whereby males reproduced the Lissajous curve with significantly less error than females, including at baseline [*F*_(1,16)_ = 10.864, *p* = 0.005, $\eta_{\text{p}}^{2}$ = 0.404; Figure 7B]. Irrespective of sex, main effects were also observed when measured across time [*F*_(7,112)_ = 8.476, *p* < 0.001, $\eta_{\text{p}}^{2}$ = 0.346]. Pairwise comparisons revealed that this increase in figural error was significant between the following timepoints when compared to baseline: 0-min (*p* = 0.017, *d* = 0.376), 1-min (*p* = 0.042, *d* = 0.280), 2-min (*p* = 0.033, *d* = 0.220). However, from 4-min post-task termination and onward, the differences in figural error were no longer significantly higher than baseline values (*p* > 0.05). Table 2 demonstrates all absolute values for MVC force, tracking error, longitudinal tracking error, normal tracking error, figural error, and the jerk ratio.

**Table 2:** All absolute values for MVC force, tracking error, longitudinal tracking error, normal tracking error, figural error, and the jerk ratio.

**Performance metric**	**Sex**	**Session**	**Baseline**	**0 min**	**1 min**	**2 min**	**4 min**	**6 min**	**8 min**	**10 min**
**MVC (N)**	Females^†^	Ext.	112.50 (23.03)	–	–	89.25 (15.29)^*^	–	95.75 (17.09)^*^	–	97.63 (17.74)^*^
		Flex.	107.75 (14.22)	–	–	92.00 (13.06)^*^	–	97.13 (16.43)^*^	–	95.50 (13.55)^*^
	Males^†^	Ext.	169.50 (42.70)	–	–	128.20 (32.36)^*^	–	137.30 (31.80)^*^	–	142.30 (31.51)^*^
		Flex.	183.50 (32.82)	–	–	139.20 (27.15)^*^	–	148.40 (29.96)^*^	–	151.00 (27.97)^*^
**Tracking error (°)**	Females^†^	Ext.	1.552 (0.234)	1.997 (0.486)^*^	1.765 (0.336)	1.634 (0.252)	1.774 (0.314)	1.621 (0.236)	1.703 (0.188)	1.705 (0.242)
		Flex.	1.609 (0.322)	2.141 (0.462)^*^	1.774 (0.398)	1.733 (0.335)	1.801 (0.228)	1.671 (0.188)	1.684 (0.352)	1.709 (0.351)
	Males^†^	Ext.	1.168 (0.182)	1.735 (0.625)^*^	1.465 (0.414)	1.321 (0.140)	1.349 (0.212)	1.315 (0.325)	1.238 (0.317)	1.271 (0.235)
		Flex.	1.243 (0.236)	1.692 (0.740)^*^	1.526 (0.678)	1.476 (0.535)	1.373 (0.419)	1.426 (0.452)	1.318 (0.280)	1.475 (0.430)
**Longitudinal tracking error (°)**	Females	Ext.	0.298 (0.378)	0.494 (0.916)	0.552 (0.579)	0.441 (0.502)	0.492 (0.828)	0.311 (0.446)	0.508 (0.474)	0.548 (0.469)
		Flex.	0.212 (0.412)	0.298 (1.065)	0.454 (0.556)	0.210 (0.474)	0.356 (0.639)	0.295 (0.353)	0.490 (0.384)	0.295 (0.506)
	Males	Ext.	0.186 (0.213)	0.734 (0.567)	0.307 (0.427)	0.164 (0.283)	0.304 (0.391)	0.232 (0.298)	0.210 (0.260)	0.253 (0.268)
		Flex.	0.180 (0.239)	0.524 (0.786)	0.221 (0.720)	0.319 (0.581)	0.308 (0.510)	0.358 (0.582)	0.263 (0.329)	0.306 (0.475)
**Normal tracking error (°)**	Females	Ext.	0.131 (0.069)	0.077 (0.233)	0.151 (0.321)	−0.041 (0.244)	0.130 (0.246)	−0.011 (0.189)	0.241 (0.173)	−0.169 (0.349)
		Flex.	0.039 (0.135)	0.046 (0.280)	−0.152 (0.110)	−0.006 (0.200)	0.102 (0.190)	0.001 (0.204)	−0.022 (0.341)	0.011 (0.181)
	Males	Ext.	0.063 (0.102)	−0.031 (0.302)	0.019 (0.211)	0.014 (0.179)	0.059 (0.075)	−0.092 (0.225)	0.071 (0.225)	0.044 (0.234)
		Flex.	0.023 (0.115)	0.281 (0.172)	−0.028 (0.241)	0.038 (0.181)	0.119 (0.249)	0.051 (0.246)	0.048 (0.188)	0.081 (0.313)
**Figural error (°)**	Females^†^	Ext.	0.842 (0.116)	0.936 (0.157)^*^	0.959 (0.122)^*^	0.881 (0.117)^*^	0.872 (0.152)	0.854 (0.102)	0.877 (0.111)	0.910 (0.170)
		Flex.	0.864 (0.168)	0.996 (0.201)^*^	0.951 (0.170)^*^	0.974 (0.192)^*^	0.891 (0.071)	0.862 (0.084)	0.895 (0.189)	0.889 (0.185)
	Males^†^	Ext.	0.636 (0.091)	0.864 (0.245)^*^	0.753 (0.173)^*^	0.745 (0.107)^*^	0.673 (0.099)	0.701 (0.160)	0.680 (0.184)	0.667 (0.147)
		Flex.	0.661 (0.109)	0.831 (0.213)^*^	0.797 (0.197)^*^	0.757 (0.203)^*^	0.704 (0.183)	0.720 (0.143)	0.669 (0.134)	0.767 (0.187)
**Jerk ratio**	Females^†^	Ext.	140.708 (30.925)	156.496 (35.705)^*^	158.370 (38.011)^*^	140.145 (26.195)^*^	160.800 (26.967)^*^	145.762 (20.574)^*^	154.723 (28.003)^*^	143.412 (16.199)
		Flex.	128.182 (32.664)	172.450 (58.196)^*^	146.822 (45.788)^*^	175.991 (49.084)^*^	153.690 (44.852)^*^	151.105 (38.710)^*^	173.169 (43.368)^*^	146.098 (27.804)
	Males^†^	Ext.	85.462 (22.271)	101.129 (30.173)^*^	126.021 (49.663)^*^	124.215 (38.915)^*^	112.260 (31.320)^*^	109.993 (36.025)^*^	104.378 (35.921)^*^	110.195 (48.005)
		Flex.	86.574 (22.892)	124.616 (36.475)^*^	128.938 (57.839)^*^	126.382 (42.015)^*^	118.838 (43.321)^*^	115.082 (27.435)^*^	119.791 (47.304)^*^	106.483 (33.145)

*Flex. = Flexion, and Ext. = extension. All values are presented as a mean (standard deviation)*.

† * = data are significantly different between sexes*;

* * = pooled data are significantly different from baseline*.

## Discussion

Participants underwent a submaximal dynamic fatigue protocol to isolate either wrist flexion or extension muscle fatigue, and then performed a hand-tracking task with performance measures collected up to 10 min post-task termination. We found that overall tracking error, figural error and the jerk ratio (with the most profound effects in the latter two variables), were fatigue- and recovery time-dependent but not muscle type fatigue-dependent. Jerk ratio a metric of movement smoothness, did not return to baseline until 10-min post-task termination and figural error did not return to baseline until 4-min post-task termination. Mixed results were found for the individual movement directions, longitudinal components, and normal components. We also found that, independent of fatigue, males had better tracking performance than females. Males were able to track the target with greater precision and smoother movements. Overall, this study demonstrated that a submaximal dynamic wrist flexion and extension fatigue protocol similarly impaired hand-tracking accuracy, and that the observed sex differences are independent of fatigue.

### The Effects of Performance Fatigability on Tracking Metrics

We hypothesized that all performance metrics would be negatively affected as a result of the submaximal dynamic fatigue protocol. Our baseline tracking error represented a non-fatigued and optimal tracking accuracy for each participant prior to the fatigue protocol. We found that, immediately post-task termination, tracking error, figural error and the jerk ratio worsened consistently across sex. Recall, these measures quantify how well participants were able to stay on target, reproduce a set trajectory, and produce smooth movement, respectively. These findings are similar to a previous investigation from our lab involving an isometric fatiguing protocol and the same hand-tracking task (Forman D. A. et al., 2020). The most interesting result from the present study is that the jerk ratio did not return to baseline until 10-min post-task termination, and figural error did not return to baseline until 4-min post-task termination. The effects of performance fatigability from the present submaximal dynamic fatigue protocol appear to be much longer lasting than our previous maximal isometric fatigue protocol, where all tracking metrics returned to baseline levels within 1–2 min post-task termination (Forman D. A. et al., 2020). In the present study, tracking error returned to baseline after 1 min of recovery, with minor changes in the normal and longitudinal components of tracking error. Nevertheless, given the prolonged impairment in the jerk ratio and figural error, we could speculate that the kinematic strategy to track and recreate the figure changed. The dynamic novelty of the present study, which induced fatigue via concentric and eccentric contractions, likely accounts for these differences from previous isometric protocols. During an eccentric contraction, the external load overcomes the muscle-generated effort, which forcefully lengthens the muscle (muscle fibers are quasi-isometrically contracting) (Reeves and Narici, 2003). Greater tension placed on the muscle fibers during the eccentric phase may subsequently lead to impaired excitation-contraction coupling and sarcomere function (Allen, 2001; Proske and Morgan, 2001). Dynamically induced muscle impairments may be the reason we observed a more pronounced increase in jerk ratio (decreased movement smoothness) in the present study. This may also be due to the link between fatigue and reduced force steadiness by a means of impaired contractile function (Missenard et al., 2008a; Ye et al., 2015). While a majority of literature focuses on fatigue at the muscle fiber level, far less focus is on the effects of muscular fatigue at the tendon. There has been a growing emphasis on the tendon and its role in force steadiness (Johannsson et al., 2015; Jakobi et al., 2018; Smart et al., 2018). In the presence of muscular fatigue, after an acute bout of submaximal dynamic exercise, the Achilles tendon has been shown to exhibit sex-dependent behaviors, in which females exhibit greater tendon elongation and reduced tendon stiffness (Joseph et al., 2014). This may not translate directly to the upper extremity due to differing roles between the two muscle groups, however, further evidence suggests that the tendon plays a significant role in force steadiness (Smart et al., 2018). It is possible that there were tendon modifications that occurred as a result of the fatiguing protocol, although further investigation would be required. Nevertheless, if peripheral mechanisms did indeed contribute to impairments in movement smoothness and hand-tracking accuracy, it is unlikely that they were solely responsible, given that peripheral mechanisms were unlikely to have recovered in 4–10 min (Brockett et al., 1997).

Others have found that task performance is impaired after a fatiguing protocol (Jaric et al., 1999; Huysmans et al., 2008). Similar to the present study, movement kinematics have been reported to change in conjunction with the maintenance of task performance. One group found that task duration and hand-arm trajectory of a hammering task were unchanged in the presence of fatigue (induced with a combination of submaximal isometric and dynamic contractions) for the shoulder, although some kinematic variables were altered (Côté et al., 2005). Others who studied the effects of an isometric fatiguing task for the wrist extensors and triceps demonstrated similar results, where movement accuracy was similar after fatigue, but with altered kinematics (Huffenus et al., 2006). Additionally, after a shoulder fatiguing protocol, kinematic adaptation was observed for several tasks, which was likely to maintain performance outcomes (Tse et al., 2016). Changes in performance may be attributed to signal-dependent noise, which, in the context of the current study, would be considered the forces that are ineffectively contributing to the task (Missenard et al., 2008a). A potential indicator of greater signal-dependent noise is increased movement jerk, which was observed until 10-min post task termination in this study. While kinematics can change but still allow individuals to generate similar performance outcomes, it may not be ideal, and working in a fatigued state may lead to injury due to altered kinematics and potentially inefficient postures.

Joint position sense is evaluated by blinding the participant to the visual positioning of their body (or body part) and having them accurately reposition their limb using their senses, as mediated through muscle spindles (Proske and Gandevia, 2012). A link has been established between painful (nociceptive) stimuli and a decrease in joint position sense in the elbow flexors (Weerakkody et al., 2003). According to Abrahams (1986), the group III and IV afferents found in skeletal muscle contain nociceptive receptors, and it is possible that the fatiguing sensation from the fatigue protocol was discomforting enough to induce kinematic changes through this pathway, via metabolite build-up (Westerblad et al., 2002). The group III and IV afferents have been said to modify proprioceptive feedback by altering mean discharge rates and stretch sensitivities of group Ia and II muscle spindle afferents (Windhorst, 2007). Furthermore, it is possible that Renshaw recurrent inhibition is also a factor in altering proprioception through modulation of muscle spindle afferents (Windhorst, 2007). In our study, after wrist extensor fatigue, we observed a tendency for participants to be farther to the right (extension direction) of the target, which may be a result of impaired proprioceptive cueing. In the literature, the effects of fatigue on joint position sense have been quite well-established. Using a submaximal dynamic fatigue protocol and a joint positioning task, proprioception was altered in the upper extremity as observed through changes and variability in end-point position (Vafadar et al., 2012). Even more proximal fatigue, such as at the cervical extensors, showed decrements in joint position sense and proprioception in the upper limb (Zabihhosseinian et al., 2015). Similar work using the WristBot and a fatiguing protocol showed decrements in proprioceptive ability after a fatiguing task at the wrist (Mugnosso et al., 2019). Lastly, it is important to note that fatiguing movements possessing an eccentric contraction result in the most significant joint position sense impairments (Brockett et al., 1997). This may explain why figural error and movement smoothness were impaired for such a prolonged period of time when compared to our previous investigation where fatigue was induced via sustained isometric contractions (Forman D. A. et al., 2020). While figural error and movement smoothness were impaired longer, it is possible that tracking error recovered after 1-min of recovery due to visual feedback of the subject position and target (Dube and Roy, 2019). This would need to be further explored using the same task but with the removal of visual feedback.

### Lack of Flexion vs. Extension Differences

We hypothesized there would be larger deficits in the wrist extensors due to their role as joint stabilizers, their lower force-generating capacity (than the wrist flexors) and that they are active during antagonist movement. In contradiction to our previous study using an isometric fatigue protocol (Forman D. A. et al., 2020), we did not find significant differences between flexor and extensor MVC force. This finding was unusual, especially in contrast to literature stating that the wrist flexors generate significantly greater wrist moments than the wrist extensors (Hallbeck, 1994; Delp et al., 1996). There was also a lack of differences in these muscle groups for the recovery of force generation, similar to our previous work (Forman D. A. et al., 2020). Wrist flexors are reported to have greater moment-generating capacity (Delp et al., 1996; Gonzalez et al., 1997), likely due to a bigger physiological cross-sectional area (Lieber et al., 1990; Jacobson et al., 1992) compared to wrist extensors. The wrist flexors have been shown to be task-specific, such that they are less active in extension tasks, whereas extensors demonstrate consistently high muscle activity during flexion movements, and are therefore always active (Forman et al., 2019; Forman G. N. et al., 2020). This is likely due to the wrist extensor's stabilizing role (Holmes et al., 2015). Regarding wrist control, one group studied dynamic force matching and found that participants exhibited greater deviation from the target force during wrist extension compared to wrist flexion (Svendsen et al., 2011). This may be indicative of better control of the flexors during fine movements compared to the extensors, which may explain why we did not observe any specific deviations in the flexion direction after flexion fatigue. Due to the greater strength capacity, more efficient moment arms and specific movement control of the wrist flexors, we expected greater decrements in all tracking metrics for the wrist extensors, and this should be further explored.

### Central and Peripheral Mechanisms

The intensity of the contraction used to induce fatigue is a critical factor to consider when investigating the effects of muscular fatigue. The intensity of the contraction has an influence on the origin of fatigue (i.e., central or peripheral) (Gandevia, 2001; Allen et al., 2008). Peripheral changes include those at the neuromuscular junction, and further distal, which principally include perturbed cellular function, such as changes to Ca^2+^ availability. A possible mechanism may be the consequences of low frequency fatigue, in which there is a marked shift in myofibrillar Ca^2+^ sensitivity due to continuous activation at lower relative loads (hence, low frequency fatigue) (Edwards et al., 1977; Allen et al., 2008). We do not doubt the effects of peripheral fatigue, but due to the lengthened impairments we believe that it was not the sole or primary factor. On the other hand, it has been reported that central fatigue is results from sustained submaximal contractions (Taylor and Gandevia, 2008; Taylor et al., 2016; Thomas et al., 2016). Central fatigue can be characterized by fatigue-induced changes at the spinal and supraspinal levels (Gandevia et al., 1996; Gandevia, 2001). This includes modifications of feedback from muscle spindles, tendon organs, and group III and IV muscle afferents (Gandevia, 2001; Klass et al., 2008; Sidhu et al., 2017). Additionally, efferent drive from the motoneuron pool is stifled due to decreases in excitatory input, an increased inhibitory input or through intrinsic motoneuron changes (Taylor and Gandevia, 2008). Furthermore, the silent period (i.e., dampened excitability) of motor evoked potentials (via transcranial magnetic stimulation) were extended during a submaximal (15% of MVC) contraction hold, but recovered immediately upon task termination (Søgaard et al., 2006). One group found that impaired responsiveness of motoneurons was responsible for impairments in motor evoked potential amplitude, as observed during a 10-min, 25% isometric contraction (McNeil et al., 2011). We believe it is appropriate to associate our findings primarily with central changes given the repetition range observed in this study and the submaximal intensity of the contractions. In our study, MVC force worsened considerably upon task termination and did not improve throughout the entire recovery period. The diminished force-generation capacity was met with concomitant impairments in all of our tracking metrics, with the jerk ratio not returning to baseline until 10-min past task termination. Overall, we believe that the long-lasting impairments in tracking performance was a result of decreased responsiveness of motoneurons in addition to intrinsic muscle impairments.

### Sex Differences

Based on previous literature, we hypothesize that fatigue-induced decrements of tracking error would be greater in males than females. We observed a significant force decrement difference between males and females, where males experienced a greater post-task termination loss of force than females relative to baseline. Sex differences in fatigue resistance is common in the literature (Hunter and Enoka, 2001; Svendsen and Madeleine, 2010; Hunter, 2016). Our work suggests that females had a greater resistance to the fatiguing task, which is consistent with work on isometric (Hunter and Enoka, 2001; Avin et al., 2010) and dynamic fatiguing tasks (Yoon et al., 2015) of various intensities in the elbow flexors. While males demonstrated less tracking error, figural error and a lower jerk ratio than females, these differences occurred across all measurement points. Thus, fatiguing submaximal dynamic contractions did not induce unique differences in tracking metrics between sexes. A plausible explanation for the observed differences between males and females may be due to absolute strength. In a study using multiple wrist postures and a force-matching task, force steadiness (coefficient of variation of force) was negatively correlated with absolute strength (*r* = −0.49), meaning higher absolute force was correlated with a lower coefficient of variation of force. Additionally, the investigators found that overall, males were more steady than females (Brown et al., 2010). Interestingly, the investigators then controlled for MVC force and found that the relationship between absolute strength and force steadiness was present regardless of sex. They attributed the force-steadiness relationship to absolute strength (Brown et al., 2010). In our study, males had greater absolute strength than females for both wrist flexion and extension, which coincides with that hypothesis. Others found that a similar relationship may be present between absolute strength and force steadiness in other muscles, such as the tibialis anterior (Dewhurst et al., 2007), hand muscles (Christou et al., 2004), and wrist extensors (Almuklass et al., 2016). Thus, in the present study, it is possible that absolute strength differences between male and female participants contributed to the observed differences in hand-tracking accuracy.

### Limitations

We only examined the muscles of the forearm, and there is evidence to suggest that these results are muscle-group dependent; our findings cannot be extrapolated to other muscle groups (Avin et al., 2010). A sample size calculation was not conducted for this study. Therefore, statistical power should be considered when interpreting our statistical models. We used a submaximal dynamic task to induce fatigue, so our findings may not be generalized to contractions involving maximal isometric contractions (Hunter, 2016). Also, the rate limiting phase of our fatigue protocol was during the concentric phase of the repetition, therefore it may be wise to examine the effects of volitional eccentric fatigue. By using the WristBot, we examined movements in flexion/extension and radial/ulnar deviation of the wrist, however, this was done with the wrist in a neutral posture. Tracking results may be different when the wrist is placed in different postures (Mogk and Keir, 2003; Brown et al., 2010). During the original conception of the study, we aimed to employ a relative percentage resistance for the fatiguing task, however, by doing such, time to fatigue could not be controlled due to known differences in time to fatigue between sexes (Hunter, 2016), and so we focused on a higher relative resistance to evoke similar onset of fatigue between sexes. We did not measure the exact relative percentage of MVC force that each participant used for their resistance weight, but by controlling the repetition range we attempted to mitigate relative differences. If we were to calculate predicted maximums using calculations designed for bench press or leg press (Reynolds et al., 2006), the intensity used in this study would be ~60–70% of 1RM. Future research using normalized loads would be a meaningful improvement upon this work. Finally, the addition of electromyographic measurements would have been useful in examining the extent of muscular fatigue through spectral analysis, and in activation pattern changes. Additionally, in the future, we will be able to employ spectral analysis, the Dimitrov Index and root mean square amplitude of the electromyographic signal in combination with the WristBot to compute the onset of fatigue (Mugnosso et al., 2018).

## Conclusion

Despite the novelty of a submaximal dynamic fatigue protocol on hand tracking accuracy in the present study, the influence of fatigue session (wrist flexion vs. wrist extension) on hand-tracking was similar to previous isometric investigations. For most performance metrics, there were no differences between muscle groups. Results showed that tracking metrics were significantly affected by the fatigue protocol. Most interestingly, movement smoothness (jerk ratio) was impaired up until 10-min post-task termination, which may have been due to a change in tracking strategy to maintain task performance (tracking error). Regarding sex differences, males demonstrated less tracking error across most metrics, although this seems to be independent of fatigue. This work is important to help understand how task performance can be maintained in the presence of muscle fatigue and to shape possible implications for workplace environments that involve repetitive, fatiguing, precision tasks.

## Data Availability Statement

The raw data supporting the conclusions of this article will be made available by the authors, without undue reservation.

## Ethics Statement

The studies involving human participants were reviewed and approved by Research Ethics Board at Brock University (REB: #16-263). The patients/participants provided their written informed consent to participate in this study.

## Author Contributions

Experimental procedures were developed by DF, GF, MM, JZ, DB, and MH. Data was collected by RK, GF, and DF and analyzed by GF and RK. The manuscript was prepared, edited, and approved by RK, GF, DF, MM, JZ, DB, and MH. All authors contributed to the article and approved the submitted version.

## Conflict of Interest

The authors declare that the research was conducted in the absence of any commercial or financial relationships that could be construed as a potential conflict of interest.
